# Regulation of calcium in pancreatic α- and β-cells in health and disease

**DOI:** 10.1016/j.ceca.2011.11.006

**Published:** 2012-03

**Authors:** Patrik Rorsman, Matthias Braun, Quan Zhang

**Affiliations:** a Oxford Centre for Diabetes, Endocrinology and Metabolism, University of Oxford, Churchill Hospital, Oxford OX3 7LJ, UK; b Alberta Diabetes Institute, University of Alberta, Edmonton T6G 2E1, Canada

**Keywords:** Insulin, Glucagon, Ca^2+^, Ca^2+^-channels, Glucose, Diabetes

## Abstract

The glucoregulatory hormones insulin and glucagon are released from the β- and α-cells of the pancreatic islets. In both cell types, secretion is secondary to firing of action potentials, Ca^2+^-influx via voltage-gated Ca^2+^-channels, elevation of [Ca^2+^]_i_ and initiation of Ca^2+^-dependent exocytosis. Here we discuss the mechanisms that underlie the reciprocal regulation of insulin and glucagon secretion by changes in plasma glucose, the roles played by different types of voltage-gated Ca^2+^-channel present in α- and β-cells and the modulation of hormone secretion by Ca^2+^-dependent and -independent processes. We also consider how subtle changes in Ca^2+^-signalling may have profound impact on β-cell performance and increase risk of developing type-2 diabetes.

## 1 Introduction

Pancreatic islets play a central role in the systemic regulation of metabolism. They do so by secreting two hormones with opposing effects on plasma glucose concentration: insulin and glucagon, which lowers and increases plasma glucose levels, respectively. The pancreatic islets are small aggregates of endocrine cells with a diameter of 100–200 μm and consist of ∼1000 endocrine cells. The three major endocrine cells within the islets are the insulin-producing β-cells, glucagon-secreting α-cells and somatostatin-releasing δ-cells which in man comprise ∼50%, 35% and 15% of the islet cell number, respectively [1].

Diabetes mellitus is a major metabolic disorder currently affecting 5–10% of the population in the western societies [2]. There are two forms of diabetes mellitus. In type-1 diabetes, the pancreatic β-cells are destroyed and patients with this form of the disease require exogenous insulin to normalise plasma glucose levels. In type-2 diabetes (T2D), which accounts for 90% of all diabetes, the β-cells largely remain intact but insulin is not released in sufficient amounts. In both forms of diabetes, the metabolic consequences of the lack of insulin are exacerbated by oversecretion of glucagon [3,4].

Electrophysiological studies on isolated α- and β-cells from both rodent (mouse, rat and guinea pig) and human islets have revealed that they are electrically excitable and that they contain a number of voltage-dependent and -independent ion channels [5,6]. Here we will summarize α- and β-cell electrical activity, the role of the different ion channels and how action potential firing translates into increases in the cytoplasmic calcium level ([Ca^2+^]_i_) that culminates in exocytotic fusion of the hormone-containing secretory vesicles.

## 2 The consensus model for glucose-induced insulin secretion

Electrical activity from mouse pancreatic β-cells was first reported by Dean and Matthews in 1968 who impaled intact mouse islets with sharp intracellular electrodes [7]. The next 15 years or research focused on the characterization of this electrical activity and its regulation by glucose [8]. When exposed to glucose concentrations too low to evoke insulin secretion (<5 mM), the β-cell is electrically inactive and the membrane potential stable and negative (typically −70 mV or below). Elevation of glucose to concentrations above 6 mM (the threshold for insulin secretion in mice) leads to membrane depolarization and, when a certain threshold potential is exceeded (−55 mV to −50 mV), the β-cells starts firing action potentials. These normally peak at voltages below 0 mV, although overshooting action potentials are occasionally observed. At glucose concentrations between 6 and 17 mM, electrical activity is oscillatory and consists of groups of action potentials superimposed on depolarized plateaux that are separated by the repolarized (electrically silent) intervals. Glucose produces a concentration-dependent increase in the fraction active phase at the expense of the silent phase. When the glucose concentration exceeds 20 mM, electrical activity is more or less continuous.

Membrane potential recordings with sharp intracellular electrodes also allowed the effects of pharmacological agents like tolbutamide and diazoxide [9], effects of channel blockers like tetraethylammonium [10], hormones and neurotransmitters such as galanin, adrenaline and acetylcholine [11] to be documented. These studies also enabled the demonstration of electrical coupling between β-cells within the same islet [12].

However, it was not until the patch-clamp technique was applied to pancreatic islet cells in the 1980s that the ion channels underlying β-cell electrical activity could be studied under voltage-clamp control. A breakthrough was the identification glucose-sensitive K^+^-channel, postulated on the basis of radioisotopic measurements in the 1970s [13], that underlie the glucose-induced membrane depolarization [14] and the subsequent finding that it is regulated by changes in the intracellular ATP and ADP concentrations [15]. Because of its high sensitivity to intracellular ATP, this channel is now referred to as the ATP-sensitive K^+^-channel (K_ATP_-channel).

Patch-clamp measurements also allowed the characterization of the voltage-dependent Ca^2+^ and K^+^-channels involved in β-cell action potential firing [16].

Based on these findings, a consensus model for glucose-stimulated insulin secretion from mouse or rat islets was proposed [6]. In this model, glucose (via its metabolism and elevation of the cytoplasmic ATP/ADP-ratio) leads to a concentration-dependent reduction in K_ATP_-channel activity. K_ATP_-channel activity maintains a negative membrane potential in mouse β-cells and closure of these channels unmasks the depolarizing influence of an (as yet) poorly characterized conductance, which accounts for the initial depolarization up to the threshold for action potential firing. The action potentials involve activation of voltage-gated L-type Ca^2+^-channels and the associated Ca^2+^-entry leads, via elevation of the submembrane [Ca^2+^]_i_, to stimulation of Ca^2+^-dependent exocytosis of the insulin-containing secretory granules [17].

Sulphonylurea drugs (like tolbutamide and glibenclamide) by-pass glucose metabolism and closes the K_ATP_-channels by direct interaction with the channel [18] but the downstream effects are largely the same.

In addition to the stimulatory effect mediated by closure of K_ATP_-channels, glucose also augments insulin release by an effect exerted distal to the elevation of [Ca^2+^]_i_. These two effects are referred to as the triggering and amplifying effects of glucose [19]. It should be emphasized that the amplifying effect requires elevation of [Ca^2+^]_i_ but that glucose somehow increases the release competence of the secretory granules and their likelihood of undergoing exocytosis in response to a given increase in [Ca^2+^]_i_. The identity of the intracellular second messenger mediating the amplifying effect remains obscure but it is of interest that increases and decreases in ATP and ADP concentration, respectively, mimic the effect of an elevation of glucose on exocytosis [20]. Accordingly, the triggering and amplifying effects of glucose may be mediated by the same second messenger(s).

Because of the electrical coupling in mouse islets, [Ca^2+^]_i_ oscillations resulting from action potential firing in the individual β-cells are synchronized across the entire islet [21,22].

**Fig. 1:**
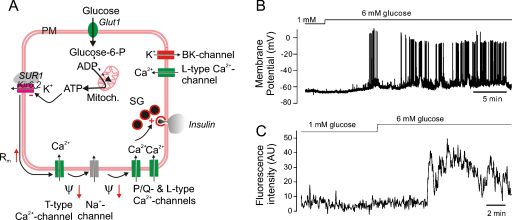
Stimulus-secretion coupling of human β-cells. (A) Glucose uptake via Glut1 leads to accelerated glucose metabolism, increased ATP production and closure of the ATP-regulated K^+^-channels (K_ATP_-channels), consisting of the pore-forming subunit Kir6.2 and the sulphonylurea-binding protein SUR1. The increased membrane resistance (*R*_m_↑) resulting from closure of the K_ATP_-channels allows occasional spontaneous opening of T-type Ca^2+^-channels to depolarise the β-cell (*Ψ*↓) and this leads to regenerative opening of additional T-type Ca^2+^-channels and further membrane depolarization that culminates in rapid activation of L-type Ca^2+^-channels and voltage-gated Na^+^-channels during the upstroke of the action potential. The action potential culminates in the opening of P/Q-type Ca^2+^-channels and the associated Ca^2+^-influx triggers exocytosis of insulin granules. Opening of Ca^2+^-activated high-conductance K^+^-channels (BK) underlies action potential repolarization. (B) Glucose-induced electrical activity recorded from a β-cell in an intact islet in response to an elevation of glucose from 1 to 6 mM. Note oscillatory electrical activity. (C) Elevation of [Ca^2+^]_i_ in a β-cell within an intact human islets when in response to an elevation of glucose from 1 to 6 mM.

Although studies on human β-cells are not as elaborate as for mouse β-cells, it seems clear that the ‘consensus model’ also applies to these cells (Fig. 1): elevation of glucose triggers electrical activity [23] and elevation of [Ca^2+^]_i_ [24]. Bursting electrical activity and [Ca^2+^]_i_ oscillations, similar (but not identical) to those seen in mouse islets, can be observed in recordings from β-cells within intact human islets (Fig. 1B–C). However, the [Ca^2+^]_i_ responses in the individual cells within human islets appear less well synchronized than in mouse islets [1,24]. This suggests that electrical coupling between individual β-cells may not be as strong in human as in mouse islets, perhaps as a result of the less organized architecture of the human islets with the β-cell ‘syncytium’ being interrupted by strands of non-β-cells [1].

## 3 The ‘lack of consensus model’ for glucose-regulated glucagon secretion

Compared to the detailed understanding of glucose-sensing in β-cells, the knowledge about this process in glucagon-secreting α-cells may seem rudimentary. Unlike β-cells, α-cells are electrically active in the absence of glucose and generate overshooting action potentials [25,26]. Surprisingly, α-cells are equipped with K_ATP_-channels with the same subunit composition as in the β-cells [27]. The role of these channels in α-cells, the presence of which was first reported in 1993 [28], has remained an enigma. How can the same channel mediate stimulation in one cell (the β-cell) and inhibition in another cell (the α-cell)? This conundrum led to the belief that these channels were perhaps not so important for the physiological regulation of glucagon secretion. This notion was supported by the finding that K_ATP_-channel activity was almost immeasurably low even in α-cells exposed to very low glucose concentrations [29,30]. Rather, the metabolic regulation of glucagon secretion was postulated to be mediated by paracrine factors released from neighbouring β- and δ-cells [5]. Potential paracrine regulators include GABA, Zn^2+^, somatostatin and insulin. However, a problem with this hypothesis is that glucagon secretion, at least in mouse islets, is regulated over a range of glucose concentrations (below 4–5 mM) not associated with any detectable stimulation of insulin and somatostatin secretion. This argues that the α-cells possess some sort of intrinsic glucose-sensing which operates independently of paracrine signalling [31,32]. The nature of the intrinsic regulation remains debated. We have proposed that K_ATP_-channels play a similar role in α-cells as in β-cells and that glucose-dependent closure of these channels results in membrane depolarization. Because α- and β-cells express different complements of voltage-gated ion channels, membrane depolarization results in reduced rather than increased electrical excitability (as in β-cells) [31].

The α-cell action potentials involve activation of TTX-sensitive voltage-gated Na^+^-channels. The significance of these channels for glucagon secretion is illustrated by the strong inhibitory effect of this blocker on glucagon secretion [33]. Voltage-gated Na^+^-channels exhibit a dual dependence on membrane potential. Whereas short depolarizations increase the open probability, sustained depolarizations (even those that are not large enough to activate the channels) make the channel enter a non-conducting inactivated state. If the depolarization is long enough, it will exert a TTX-like effect on α-cell electrical activity and glucagon secretion. We have found that depolarizations of only a few millivolts are sufficient to reduce action potential height by 10–20 mV. Such an effect can be expected to have a dramatic effect on glucagon secretion because exocytosis declines exponentially by >10% for every millivolt reduction of action potential height once their peak voltage goes below 0 mV [34].

It should be emphasized that ‘intrinsic’ and ‘paracrine’ modes of α-cell regulation are not mutually exclusive. Indeed, there is good evidence for paracrine regulation of glucagon secretion [35–38] but its role is likely to be particularly significant under conditions associated with stimulation of insulin and/or somatostatin secretion (or other factors co-released with these hormones).

## 4 Ca^2+^-channels in β-cells and their role in insulin secretion

Mouse β-cells are equipped with dihydropyridine-sensitive Ca^2+^-channels (50% of the whole-cell Ca^2+^-current), SNX482-sensitive R-type Ca^2+^-channels (25%) and P/Q-type Ca^2+^-channels (10–15%) [39]. Pharmacological blockade of L-type Ca^2+^-channels leads to transient inhibition of glucose-induced electrical activity but it subsequently resumes in the continued presence of the blocker [40]. Pancreatic islets have variably been reported to express L-type Ca^2+^-channels containing α_1C_ (Cav1.2) and α_1D_ (Cav1.3) pore-forming subunits [41,42]. However, we observed no differences in the Ca^2+^-current density between wildtype and Cav1.3/α_1D_-deficient β-cells [43]. By contrast, the Ca^2+^-current density was reduced by ∼50% in β-cells from Cav1.2^−/−^ mice and the residual current was resistant to dihydropyridines antagonists (like isradipine) and agonists (like BAYK8644) [39,44]. We therefore concluded that Cav1.2 is the principal L-type Ca^2+^-channels subtype in mouse β-cells.

Glucose-induced action potential firing and [Ca^2+^]_i_ oscillations were maintained in β-cells/islets from mice lacking Cav1.2 (α_1C_) L-type Ca^2+^-channels. Nevertheless, glucose-induced insulin secretion is strongly reduced in Cav1.2^−/−^ islets. Glucose-induced insulin secretion follows a biphasic time course consisting of a rapid but transient 1st phase followed by a slower and sustained 2nd phase [17]. Both phases were reduced in Cav1.2-deficient islets [39]. Thus, Ca^2+^-influx via α_1C_-containing L-type Ca^2+^-channels is tightly linked to insulin exocytosis.

Experiments on islets from mice lacking R-type Ca^2+^-channels (Cav2.3/α_1E_ knock-out mice) and the impact of the R-type Ca^2+^-channel blocker SNX482 indicate these Ca^2+^-channels may play a role in the supply of new secretory granules to the release sites. This is suggested by the observation that whereas 1st phase insulin secretion was unaffected by pharmacological/genetic inhibition of R-type Ca^2+^-channels, 2nd phase secretion was strongly reduced [45].

There are some important differences between human and mouse β-cells in terms of Ca^2+^-channel expression [46]. First, insulin exocytosis in human β-cells shows a strong dependence on P/Q-type Ca^2+^-channels. Second, human β-cells are equipped with T-type Ca^2+^-channels that start activating at membrane potentials as negative as −60 mV. Third, R-type Ca^2+^-channels are not at all expressed in human islets.

In mouse β-cells, the Ca^2+^-currents can be modulated by protein phosphorylation: activation of PKA and PKC leads to slight (20%) increase in Ca^2+^-entry [47,48]. However, the effects of physiological agonists (like GLP-1 and acetylcholine) acting via these pathways are in general much weaker [49,50]. There is evidence suggesting that the Ca^2+^-channels are subject to stronger modulation by agonists in rat β-cells [51] and in human β-cells (own unpublished). Nevertheless, it seems justified to conclude that most of the hormonal modulation of insulin secretion (inhibition or stimulation) is exerted at the level of exocytosis itself.

## 5 Ca^2+^-channels in α-cells

The first characterization of α-cell Ca^2+^-currents was performed using guinea pig α-cells and indicated that they contain low-threshold T-type Ca^2+^-channels and two types of high voltage-activated (HVA) Ca^2+^-channels [52]. More recent work suggests that mouse α-cells express a similar complement of Ca^2+^-channels [26,53,54]. We proposed, based on the use of nifedipine and ω-conotoxin that mouse and rat α-cells contain L- (80%) and N-type HVA Ca^2+^-channels (20%) [55]. However, the effect of ω-conotoxin was reversible, unlike what has been demonstrated for its interaction with N-type Ca^2+^-channels, suggesting that the effect of ω-conotoxin in α-cells may be unspecific [56]. Indeed, mouse α-cells express only low levels of Cav2.2 (N-type) but high levels of Cav2.1 (P/Q-type) (own unpublished). It is possible therefore that mouse α-cells express P/Q- rather than N-type Ca^2+^-channels in addition to L-type Ca^2+^-channels. We have subsequently confirmed that α-cells contain an HVA Ca^2+^-current component that can be blocked by the P/Q-type Ca^2+^-channel blocker ω-agatoxin and that ω-conotoxin exerts no additive inhibitory effect in α-cells already exposed to ω-agatoxin (own unpublished).

**Fig. 2:**
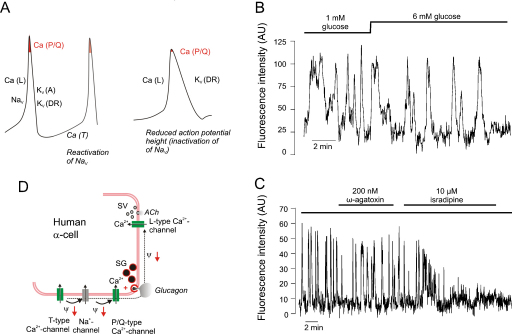
Ca^2+^-signalling in human α-cells. (A) Schematic action potential in human α-cell. Contribution to action potential of T- (Ca[T]), L- (Ca[L]) and P/Q-type Ca^2+^-channels (Ca[P/Q]), TTX-sensitive Na^+^-channels (Na_V_), transient A-type K^+^-current (K_V_[A]) and delayed rectifying K^+^-current (K_V_[DR]). P/Q-type Ca^2+^-channels, linked to glucagon exocytosis, open only at the peak of the action potential (highlighted in red) and if the peak voltage of the action potential is reduced (right), fewer P/Q-type Ca^2+^-channels will open with resultant suppression of glucagon secretion. Glucose may reduce spike height via membrane depolarization and this in turn leads to voltage-dependent inactivation of Na_V,_ K_V_(A) and Ca(T) and under these conditions, action potential firing may depend only on Ca(L) and K_V_(DR) channel activity. (B) Effects of increasing glucose from 1 to 6 mM on spontaneous [Ca^2+^]_i_ oscillations in an individual cell (assumed to be an α-cell) within an intact human pancreatic islet. Note that glucose has no major inhibitory effect during >10 min. (C) Effects of ω-agatoxin (200 nM) and isradipine (10 μM) on [Ca^2+^]_i_ measured in a cell spontaneously active at low glucose. Note small effect of ω-agatoxin and that subsequent addition of isradipine exerts a stronger inhibitory effect. Effect of ω-agatoxin irreversible so both P/Q and L-type Ca^2+^-channels are blocked following the addition of isradipine. Experiments in B–C were conducted by Dr CE Ward. Trace in C is taken from [34]. (D) Differential roles (hypothetical) of L- and P/Q-type Ca^2+^-entry on release of glucagon-containing secretory granules and acetycholine-containing synaptic like microvesicles (SV). Note that L- and P/Q-type Ca^2+^-channels are spatially separated.

Human α-cells are also equipped with P/Q- and L-type Ca^2+^-channels [34]. However, their relative contribution of L- and P/Q-type Ca^2+^-channels to Ca^2+^-entry is opposite to that in mouse α-cells (P/Q-type contributing 70% and L-type only 20%). Like their rodent counterparts, human α-cells contain low-threshold T-type Ca^2+^-channels that may be involved in the pace-making of the α-cell (i.e. the interspike depolarization between two successive action potentials). Fig. 2A depicts schematically how the voltage-gated ion channels documented in human α-cells contribute to different phases of the action potential.

## 6 Economical use of Ca^2+^-channels in β-cells

The Ca^2+^-channel density in β-cells is very low (10 pA/pF measured during depolarizations to 0 mV in the presence of 10 mM Ca^2+^) [16]. With a single-channel current amplitude of 0.2 pA (at 0 mV with 10 mM Ca^2+^) [57] and a specific membrane capacitance of 10 fF μm^−2^ [58], the above current density corresponds to 0.5 Ca^2+^-channels per μm^2^. A similar low Ca^2+^-channel density was derived by stationary fluctuation analysis [43]. This is only 5–10% of the channel density in adrenal chromaffin cells but apparently enough to allow the β-cell to secrete adequate amounts of insulin. Clearly, the β-cell has developed a means to ensure efficient usage of the limited amounts of Ca^2+^ entering the cell. It is noteworthy that although [Ca^2+^]_i_ remains elevated for a considerable period after a membrane depolarization, exocytosis only proceeds during the depolarization, echoing the activity of the Ca^2+^-channels [59]. This behaviour indicates that exocytosis in the β-cell is controlled by [Ca^2+^]_i_ just below the inner mouth of the Ca^2+^-channels. It is implicit from this concept that only secretory granules within these ‘active zones’ of elevated [Ca^2+^]_i_ (<10 nm from the inner mouth of the Ca^2+^-channels) will undergo exocytosis. The finding that rapid exocytosis is resistant to intracellular application of 10 mM EGTA is consistent with this scenario [60]. Upon membrane repolarization and Ca^2+^-channel closure, the active zones almost instantly collapse and this accounts for the rapid termination of exocytosis.

**Fig. 3:**
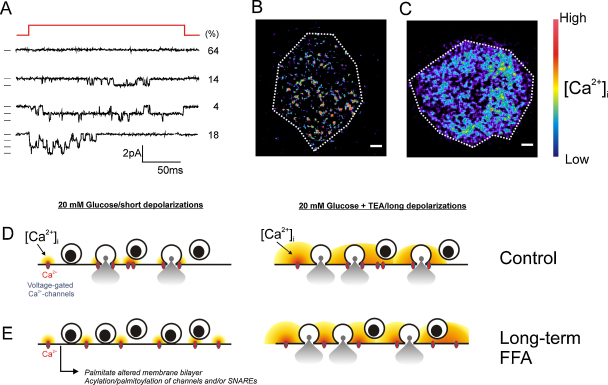
Uneven Ca^2+^-channel distribution in β-cells. (A) On-cell recording of Ca^2+^-channel activity in a single mouse β-cell showing examples of patches containing no, 1, 2 or 3 channels. Values to the right indicate probability (in %) of the respective types of responses. Data from [43]. (B) TIRF image of an isolated ‘control’ β-cell during a 50-ms depolarization from −70 mV to 0 mV. Spatial resolution was increased by the inclusion of 10 mM EGTA in intracellular medium (to restrict Ca^2+^-diffusion) and use of the low-affinity Ca^2+^-indicator Oregon Green 6F (30 μM). Changes in [Ca^2+^]_i_ are displayed in pseudocolours with black/blue and yellow/red corresponding to very low and high concentrations, respectively. Scale bars: 2 μm. The dotted line corresponds to the approximate footprint of the β-cell. (C) As in B but obtained in a β-cell treated for 72 h with 0.5 mM palmitate. Experiments in panels B–C were conducted by Dr MB Hoppa. Data from [60]. (D) Schematic representation of Ca^2+^-channels and secretory granules in control cells. Secretory granules are tightly associated with voltage-gated Ca^2+^-channels. Stimulation with glucose leads to membrane depolarization, opening of voltage-gated Ca^2+^-channels, localized increases in [Ca^2+^]_i_ close to Ca^2+^-channels. This triggers exocytosis of the secretory granules. During long depolarizations (or when TEA was applied to broaden the action potentials), the active zones of elevated [Ca^2+^]_i_ extend further away from the Ca^2+^-channels leading to moderate (50%) further stimulation of secretion. (E) As in D but in palmitate-treated β-cells in which Ca^2+^-channels and secretory granules are not so tightly associated. Under these conditions, the [Ca^2+^]_i_ increases occur too far away from secretory granules to trigger their release. However, insulin secretion can be rescued when the duration of the depolarizations is increased (e.g. by TEA). Under these conditions, the size of the active zones is increased so that also granules not situated close to the Ca^2+^-channels undergo exocytosis.

On-cell single-channel measurements of Ca^2+^-channel activity in mouse β-cells have revealed that whereas most (>60%) membrane patches contain no active Ca^2+^-channels at all, ∼20% of the membrane patches contained 3 active channels [43] (Fig. 3A). This is not expected from random distribution of the Ca^2+^-channels and instead argues that the β-cell Ca^2+^-channels associate into triplets. This conceivably cancels out stochastic variation of channel activity and ensures that a release-competent secretory granule residing close to the Ca^2+^-channels will be exposed to exocytotic levels of [Ca^2+^]_i_ long enough to trigger secretion. It is interesting that TIRF imaging of the near-membrane [Ca^2+^]_i_ transients evoked by brief depolarizations concentrate to a few discrete areas (Fig. 3B). It is tempting to speculate that these correspond to the triplets of Ca^2+^-entry seen in the single-channel measurements.

In β-cells in intact mouse pancreatic islets, the maximum rate of exocytosis during depolarization to 0 mV is 30–40 fF/s [61]. This corresponds to the secretion of 10–15 granules/s. When taking into account that the β-cell action potentials peak at −20 mV, the steep voltage dependence of exocytosis and that the measurements were conducted in the presence of high intracellular cAMP, the rates of exocytosis indicated by capacitance measurements are in reasonable agreement with those observed in biochemical measurements of insulin secretion (∼0.25 granule/s and cell) [17].

The relationship between the duration of the stimulus and the exocytotic response is almost linear. This observation raises the interesting possibility that biphasic glucose-induced insulin results from the time-dependent changes in electrical activity and [Ca^2+^]_i_ following a step elevation of glucose [62] rather than the sequential release of distinct functional pools of secretory granules that differ with regard to release competence and/or proximity to the release sites [17]. This scenario may also be in better agreement with recent TIRF imaging data suggesting that most granules released in response to glucose are ‘restless newcomers’, not residing at the plasma membrane but that arrive at the plasma membrane during glucose stimulation and then promptly undergo exocytosis [63]. It is tempting to speculate that the release sites correspond to areas of the plasma membrane where L-type Ca^2+^-channels congregate, the only place in the β-cell where the submembrane [Ca^2+^]_i_ is likely to rise to exocytotic levels. This scenario is supported by the finding that 1st phase insulin secretion evoked by an increase in glucose from 3 to 22 mM is maintained in mouse islets loaded with the fast Ca^2+^-buffer BAPTA to an estimated intracellular concentration of 1 mM [64]. By contrast, 2nd phase insulin secretion was abolished, indicating that it depends on a more global elevation of [Ca^2+^]_i_.

Membrane depolarization alone without concomitant stimulation of β-cell metabolism (e.g. using high-K^+^ or tolbutamide in the complete absence of glucose) evokes only a transient stimulation of insulin secretion reminiscent of 1st phase glucose-induced insulin secretion [65]. Ultrastructural [66] as well as TIRF [60] and confocal [67] live-cell imaging experiments suggest that insulin secretion evoked by this stimulation paradigm, unlike that evoked by glucose [68], to a large extent involves granules already docked below the plasma membrane. Once this pool of docked granules has been depleted, and in the absence of any supply of new granules for release, exocytosis stops and this explains the transient nature of insulin secretion under these experimental conditions.

Collectively, the observations summarized above suggests that insulin secretion evoked by membrane depolarization alone (e.g. in response to elevated extracellular K^+^) and that elicited by glucose result in the release of distinct pools of secretory granules. This raises the interesting question of how the secretory granules can distinguish between Ca^2+^ entering the cell in response to the two different stimuli. Factors that may be relevant in this context include the nature of the electrical stimulus (sustained depolarization *vs*. brief action potentials) as well as biochemical effects influencing the release probability of the secretory granules.

The existence of biochemically distinct population of secretory granules is supported by the recent and elegant FRET-based studies by Takahashi et al. [69]. These authors demonstrate that the SNARE complexes in β-cell exist in different states of ‘pre-assembly’. Granules with pre-assembled SNARE complexes (high-FRET) undergo rapid exocytosis. Tentatively, this subset of granules corresponds to those that undergo exocytosis in response to depolarization alone. In addition, a time- and Ca^2+^-dependent assembly of SNARE complexes was observed that preceded slower exocytosis, that may represent the mobilization of new granules for release. Finally, they observed rapid assembly of SNARE complexes preceding ‘crash fusion events’ (i.e. newcomer release) triggered by glucose.

## 7 Ca^2+^-dependent exocytosis in α-cells: role of L- and non-L-type Ca^2+^-channels

**Fig. 4:**
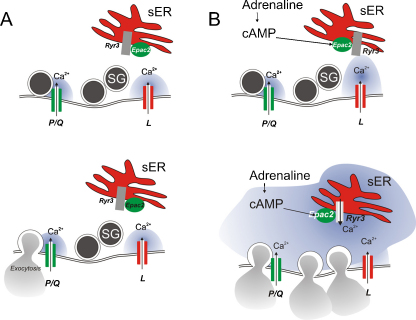
Ca^2+^-channels and glucagon exocytosis evoked by hypoglycaemia alone and in the presence of adrenaline. (A) Action potential generated under hypoglycaemic conditions leads to opening of non-L-type (presumably P/Q-type) Ca^2+^-channels and localized increases in [Ca^2+^]_i_ that trigger exocytosis of the few glucagon-containing secretory granules (SG) that happen to be situated close enough to these Ca^2+^-channels. During the action potentials, L-type Ca^2+^-channels also open but exocytosis of secretory granules is not triggered (spatial separation?). (B) In the presence of adrenaline, cAMP increases and activates the cAMP-sensor Epac2. Under these conditions, Ca^2+^-entry via L-type Ca^2+^-channels triggers Ca^2+^-induced Ca^2+^-release by activation of ryanodine receptor Ca^2+^-release channels (RyR3) in the sER and [Ca^2+^]_i_ rises throughout the α-cell triggering exocytosis of all release-competent granules.

During α-cell electrical activity both L- and non-L-type Ca^2+^-channels (N- or P/Q-type; see discussion above) open. In mouse α-cells, L-type Ca^2+^-channels mediate most of the Ca^2+^-entry during the action potentials but glucagon secretion evoked by low glucose alone is almost resistant to isradipine or nifedipine [29,70,71]. Conversely, blocking non-L-type Ca^2+^-channels, whilst having only a weak effect on [Ca^2+^]_i_, inhibits glucagon secretion as strongly as elevation of glucose to >6 mM [71]. This suggests that α-cells remain capable of generating action potentials even when the L-type Ca^2+^-channel activity is completely suppressed. In this context it may be pertinent that α-cells in freshly isolated mouse islets are equipped with voltage-gated Na^+^-channels and that the Na^+^-current amplitude is 5–10-fold larger than all the Ca^2+^-currents together. During these action potentials, non-L-type Ca^2+^-channels may still open and trigger exocytosis of the glucagon vesicles. However, it remains obscure why Ca^2+^-entry via L-type Ca^2+^-channels is such a poor initiator of glucagon secretion although L-type Ca^2+^-channels outnumber non-L-type Ca^2+^-channels by a factor of four. Perhaps the simplest explanation is that L- and non-L-type Ca^2+^-channels are spatially segregated so that their active zones do not overlap (Fig. 4A). Because non-L-type HVA Ca^2+^-channels only open during the peak of the action potential, a reduction of action potential height can (as discussed above) be expected to have dramatic effects on glucagon secretion. This explains why tolbutamide inhibits glucagon secretion from both mouse and human islets [32]. Application of tolbutamide to α-cells leads to strong membrane depolarization and (via voltage-dependent inactivation of the Na^+^-channels) to a marked reduction of spike height [32,34]. Thus, even at the peak of the action potential the non-L-type Ca^2+^-channels do not activate sufficiently to trigger exocytosis (Fig. 2A). We are currently investigating whether glucose exerts a tolbutamide-like effect on α-cell electrical activity. If this turns out to be the case, it would explain how elevation of glucose inhibits glucagon secretion without a detectable decrease in [Ca^2+^]_i_ in α-cells in intact mouse [72] and human islets (Fig. 2B). Indeed, the blocker of P/Q-type Ca^2+^-channels has only a small effect on [Ca^2+^]_i_ in spontaneously active α-cells in human islets exposed to 1 mM glucose and yet strongly inhibits glucagon secretion [34]. If elevation of glucose, via reduced action potential amplitude, leads to a similar reduction of P/Q-type Ca^2+^-channel activity, then this effect would be equally difficult to detect in [Ca^2+^]_i_ measurements.

Collectively, these observations are suggestive of a dichotomy between [Ca^2+^]_i_ and glucagon secretion. It is tempting to speculate that Ca^2+^-entry via L-type Ca^2+^-channels at elevated glucose is linked to cellular processes other than glucagon secretion. It was recently reported that human α-cells secrete acetylcholine and it was proposed that this, via activation of muscarinic receptors, increases the responsiveness of neighbouring β-cells to increases in glucose [73]. However, if acetylcholine was secreted by the same mechanism as glucagon, then its release (like glucagon) would be suppressed when glucose is elevated (i.e. under conditions where it was postulated to enhance insulin secretion). Thus, Ca^2+^ entry via two different Ca^2+^-channels may allow two exocytotic pathways to operate in parallel within the α-cell (Fig. 2D).

Whereas L-type Ca^2+^-channels play only a minor role in glucagon secretion evoked by low glucose alone, the situation changes dramatically when glucagon secretion is stimulated by adrenaline [74]. The stimulation of glucagon secretion by adrenaline is an important component of the counter-regulatory response of the α-cell to hypoglycaemia, which becomes defective in diabetic patients [3]. It could be argued that understanding the processes involved in counter-regulation in the healthy α-cell provides the knowledge base necessary to pharmacologically restore it in the diabetic α-cell. Intriguingly, the stimulation of glucagon secretion by adrenaline is associated with a switch in the Ca^2+^-channel dependence from non-L-type to L-type Ca^2+^-channels [55,74]. Thus, glucagon secretion evoked by adrenaline is strongly inhibited by isradipine or nifedipine.

The stimulatory effect of adrenaline on glucagon secretion is mediated by cAMP by a PKA-independent mechanism that involves the cAMP-sensing protein Epac2 [55,74]. We previously proposed that this recruits secretory granules to the L-type Ca^2+^-channels [53]. However, more recent experiments (own unpublished) suggest that Ca^2+^-entry via L-type Ca^2+^-channels when Epac2 is activated (by high intracellular cAMP) promotes Ca^2+^-induced Ca^2+^-release (CICR) from intracellular Ca^2+^ stores. We speculate that the resulting global elevation of [Ca^2+^]_i_ within the α-cell triggers exocytosis of any release-competent secretory granules regardless of their proximity to the Ca^2+^-channels (Fig. 4B) and this accounts for the massive stimulation of glucagon secretion.

## 8 Ca^2+^-channels, [Ca^2+^]_i_ and diabetes

As should be evident from the discourse above, Ca^2+^-channels and [Ca^2+^]_i_ are intimately linked to the both insulin and glucagon secretion. Is this information relevant to the understanding of the pathophysiology of type-2 diabetes?

Whereas acute exposure of pancreatic islets to free fatty acids results in stimulation of insulin secretion [75], protracted exposure (48 h or more) impairs glucose-induced insulin secretion [76]. The underlying mechanism has been difficult to unravel and it has been attributed to impaired metabolism, hyperactivation of K_ATP_-channels and even reduced β-cell mass due to increased β-cell death [77,78].

With the fairly detailed understanding of the stimulus-secretion coupling of the pancreatic β-cell we thought it would be straightforward to pinpoint the changes produced by long-term exposure to FFAs such as palmitate and oleate. Surprisingly, β-cells exposed to FFAs for 72 h performed better than control cells not exposed to FFAs when key functional parameters like glucose metabolism (ATP production), K_ATP_-channel closure, glucose-induced elevation of [Ca^2+^]_i_, Ca^2+^-currents and exocytosis (measured in response to 500-ms depolarizations) were analyzed. Likewise, there were no signs of increased β-cell death or reduced insulin content/biosynthesis that would explain the reduction of glucose-induced insulin secretion [76].

We subsequently discovered that long-term exposure to FFAs results in a selective suppression of exocytosis elicited by brief (action potential-like) depolarizations. Thus, exocytosis in response to 50-ms depolarizations was strongly reduced. By contrast, consistent with our earlier data [76], exocytosis evoked by longer depolarizations (>300 ms) was not affected. This selective suppression of exocytosis evoked by short (action potential-like) depolarizations correlated with dispersion of Ca^2+^-entry. Whereas depolarization-induced Ca^2+^-entry is concentrated to discrete areas in control cells (Fig. 3B), it becomes more diffuse in β-cells exposed to FFAs (Fig. 3C). Because of the loss of Ca^2+^-channel aggregation, release-competent granules may not be exposed to exocytotic levels of [Ca^2+^]_i_ during the brief action potentials of the β-cells (Fig. 3D–E). However, insulin secretion can be rescued by prolonging the duration of the voltage-clamp depolarizations to >300 ms. During such long depolarizations, there is enough time for Ca^2+^ to diffuse within the cell to produce a global elevation of [Ca^2+^]_i_ that eventually triggers exocytosis also of granules that are not situated in the immediate vicinity of the Ca^2+^-channels.

Long-term FFA exposure also interfered with glucose-stimulated insulin secretion from human islets. In both mouse and human islets [60], the suppression of glucose-induced insulin secretion resulting from chronic FFA exposure could be reversed by addition of the broad-spectrum K^+^-channel blocker TEA. This compound blocks the K^+^-channels involved in action potential repolarization and thereby extends the duration of the action potentials. This allows the Ca^2+^-channels to stay open longer (mimicking the effects of long voltage-clamp depolarizations), resulting in a more global elevation of [Ca^2+^]_i_ than in response to brief action potentials. Thus, Ca^2+^-channel dispersion may underlie the suppression of glucose-induced insulin secretion by long-term FFA exposure also in human islets.

The functional consequences of Ca^2+^-channel dissociation may be particularly strong in the β-cell because it operates with so few Ca^2+^-channels. In cells with a higher Ca^2+^-channel density (like chromaffin cells), dispersion of the Ca^2+^-channels and dissociation of the granule/Ca^2+^-channel complexes can be expected to have a much smaller effect because the secretory granules will almost always be situated in the close vicinity of one or several voltage-gated Ca^2+^-channels.

What is the physiological significance of Ca^2+^-channel dispersion in response to long-term FFA exposure? We propose that this provides the β-cells with a means to suppress insulin secretion during fasting/starvation. Under these conditions, plasma FFA increases to >1 mM whilst plasma glucose, by activation of glucogenic mechanisms, is maintained >3.5 mM [79]. Normally, the combination of this glucose concentration and FFAs would lead to strong stimulation of insulin secretion. This is clearly inappropriate during starvation when metabolism should be geared towards mobilization of glucose rather than its disposal. It is possible that this evolutionarily preserved mechanism, which originally developed to suppress insulin secretion during food deprivation, becomes erroneously activated when plasma and/or intra-islet levels become elevated via excessive dietary intake of lipids [80]. It is easy to see how this can lead to a vicious cycle of progressive impairment of insulin secretion and elevation of plasma FFA levels until overt type-2 diabetes presents in genetically predisposed individuals [81].

Because of limited availability of islets from donors with type-2 diabetes (T2D), the impact type-2 diabetes on β-cell [Ca^2+^]_i_ remains unknown. However, work on β-cells from GK rats, a model of non-obese human T2D with elevated plasma FFA levels [82], found no impairment in the capacity of glucose to elevate [Ca^2+^]_i_ and only subtle changes in exocytosis manifested as a ∼50% reduction of the Ca^2+^-sensitivity of exocytosis [83]. The dispersion of Ca^2+^-channels we observe following long-term FFA exposure can be expected to result in an apparent reduction of the Ca^2+^-sensitivity of exocytosis but whether this underlies the diminished insulin secretion in GK-rats (and in islets from T2D donors) remains to be demonstrated experimentally.

## 9 Coda

It is rewarding to compare Ca^2+^-signalling in pancreatic α- and β-cells. Such studies may reveal processes that are key to the reciprocal glucose-sensing in the two cell types. It is also important to note that there are numerous and important differences between human and rodent islet cells. This illustrates the potential pitfalls of uncritically extrapolating observations made in rodent systems to the situation in man. Finally, there appears to be some truth to the adage that “you are what you eat”, at least when it comes to Ca^2+^-signalling in the β-cell. As we have discussed above, diets that lead to protracted exposure of the β-cells to FFAs may exert adverse effects on the β-cell's capacity to secrete insulin in response to glucose.

## Conflict of interest

The authors declare that there are no conflicts of interest.
